# Editorial: Mitochondrial dysfunction as a target in neurodegenerative diseases

**DOI:** 10.3389/fnmol.2023.1271175

**Published:** 2023-09-12

**Authors:** Lorena Perrone

**Affiliations:** Faculty of Medicine, University of Enna “Kore”, Enna, Italy

**Keywords:** mitochondria, neurodegeneration, cell-specific, microglia, mitophagy, DRP1, sex-specific, proteomics

The factors underlying the onset and progression of neurodegeneration are not yet fully elucidated, resulting in difficulties for the development of therapeutic strategies aimed to prevent, slow down and cure neurodegenerative diseases (Blagov et al., 2023). It is emerging a key role of mitochondrial dysfunction in the pathogenesis of several neurodegenerative diseases (Alqahtani et al., 2023). Due to their long life and high energy demand, neurons are very sensitive to energy imbalance and oxidative stress, which are modulated by mitochondria. In addition, neurons are characterized by a highly polarized structure, which underlines the presence of specific subcellular domains that play specific function. Thus, neurons need to deliver mitochondria through long axons and terminal branches that are characterized by specific energy demands (Yang et al., 2023). Although it is well accepted the role of mitochondria dysfunction in promoting neurodegeneration, it is difficult to define disease-specific mitochondria alterations, due to the multitude of function regulated by mitochondria.

In this special issue, we present article shedding new insight on specific functions played by mitochondria and that are implicated in neurodegeneration.

Mitochondrial proteins regulate a large number of mitochondrial functions, including mitochondria bioenergetics, mitochondrial DNA maintenance, import machinery, ion channels as well as mitochondrial morphogenesis and dynamics (Pfanner et al., 2019). In humans mitochondria contain at least 1500 proteins (Lefort et al., 2009). Mitochondria contain their own DNA encoding and synthetizing 13 specific proteins that are all subunits of the respiratory chain. The other mitochondrial proteins, about 99%, are encoded by the nuclear DNA, synthetized in the cytoplasm and imported into mitochondria. Notably, the function of about 20% of mitochondrial proteins is still unknown. Considering the high relevance of mitochondria protein in modulating the mitochondrial function, the first article of this Research Topic “*Mitochondrial protein dysfunction in the pathogenesis of neurological diseases*” (Wang et al.) summarizes the discoveries about the mitochondrial proteomes, underlying the effect of aberrant expression of mitochondrial proteins in promoting neurodegeneration and providing a clear review correlating specific mitochondrial protein alterations and their role in defined pathways that affect neuronal function.

Altered mitochondrial dynamics and enhanced mitochondrial fragmentation are also involved in neurodegeneration (Bhatti et al., 2023). Recent studies demonstrate the role of dynamin-related protein 1 (Drp1), a protein involved in mitochondria fission, in promoting neurodegeneration, especially in Alzheimer's disease (AD) (Bhatti et al., 2023). Altered Drp1 affects mitochondrial morphology, dynamics and bioenergetics in neuronal cells (Bhatti et al., 2023). However, recent data demonstrate that Drp1 may affects other cell types other that neurons, still promoting neurodegeneration by promoting the activation of the NLRP3 inflammasome (Zhang et al., 2020; Sbai et al., 2022), suggesting that mitochondrial dysfunction-induced inflammation is implicated in neurodegeneration. The second article of this Research Topic “*Is Drp1 a link between mitochondrial dysfunction and inflammation in AD?*” (Sbai et al.) summarizes the recent findings demonstrating the key role of Drp1-induced mitochondrial dysfunction in promoting the activation of the NLRP3 inflammasome, leading to inflammation and contributing to neurodegeneration in AD. These data underline the role of mitochondria in promoting inflammation, showing that inflammation plays a key role in promoting neurodegeneration in AD.

Mitochondria quality control is essential for the maintenance of mitochondrial health. Mitophagy plays a key role in this maintenance, by delivering damaged mitochondria to lysosomes for degradation. Thus, impaired mitophagy leads to the accumulation of damaged mitochondria, promoting neurodegeneration (Li et al., 2023). PINK/Parkin regulates the mitophagy process, which in turn modulates the initiation of autophagy (Li et al., 2023). Alterations in the mitophagy process play a key role in prion diseases (Li et al., 2023). Prion diseases are a class of severe and lethal neurodegenerative diseases due to the infection of the pathogenic form of the prion protein (PrP), which is misfolded and produces pathologic aggregates (Maddox et al., 2020). The third article of this Research Topic “*Cardiolipin externalization mediates prion protein (PrP) peptide 106-126-associated mitophagy and mitochondrial dysfunction*” (Yang et al.) is a research articles shading new insights on the role of PrP in affecting mitophagy. This work unveils the relevance of a new pathway. The authors demonstrate that PrP^106 − 126^ peptide induces the externalization of cardiolipin (CL), a mitochondria-specific phospholipid in neuronal cells. CL externalization in turn plays a role in mitophagy, by interacting with LC3II at the outer membrane of mitochondria. Notably, CL inhibition decreases PrP^106 − 126^ -induced mitophagy, decreases PINK1 and Drp1 recruitment to mitochondria, affects the oxidative phosphorylation and promotes oxidative stress, leading to mitochondrial dysfunction. These data shed new lights on the role of PrP^106 − 126^ in non-ubiquitination-mediated mitophagy, opening the way for the discovery of new small molecules as modulators of mitophagy.

Alterations in mitochondrial respiration are observed in neurodegenerative diseases. As mentioned above, it is crucial to understand the cellular cell type involved in mitochondrial dysfunction-driven neurodegeneration. Although mitochondria play a key role in neuronal cells, they affect the function of other cell type, which in turn can promote neurodegeneration. Notably, gender-specific alterations have been suggested and this point is important for the diagnosis and cure of neurodegenerative diseases, with particular regard to AD (Castro-Aldrete et al., 2023). Thus, it is necessary to develop a model suitable for the understanding of cell-specific and gender-dependent mitochondrial alterations that play a specific role in neurodegeneration. Induced pluripotent stem cells (iPSCs) derived from human fibroblast may help in the study of cell-specific and gender-specific differences in mitochondrial function and contribution in the progression of neurodegenerative diseases, such as AD. The fourth article of this Research Topic “*Cell type and sex specific mitochondrial phenotypes in iPSC derived models of Alzheimer‘s Disease*” (Flannagan et al.) is an original research article demonstrating the relevance of cell-specific and sex-specific differences in mitochondrial dysfunction occurring in AD. This study underlined the differences between astrocytes and neurons in mitochondrial dysfunction. Notably, this study demonstrates the relevance of sex-specific alterations in mitochondrial dysfunction in AD, shedding new light for a better diagnosis of AD and the development of therapeutics.

In conclusion, all the articles of this Research Topic focus on emerging and innovative studies analyzing new mitochondrial-related pathways involved in neurodegeneration (Figure 1). Indeed, it sheds new light on the role of the mitochondrial proteome in neurodegeneration, as well as demonstrates the essential function of Drp1 in promoting both mitochondrial dysfunction in microglia and mitophagy. This Research Topic underlines that mitochondria dysfunction-induced neurodegeneration involves cell-specific pathways and mitochondrial alterations are also sex-specific.

**Figure 1:**
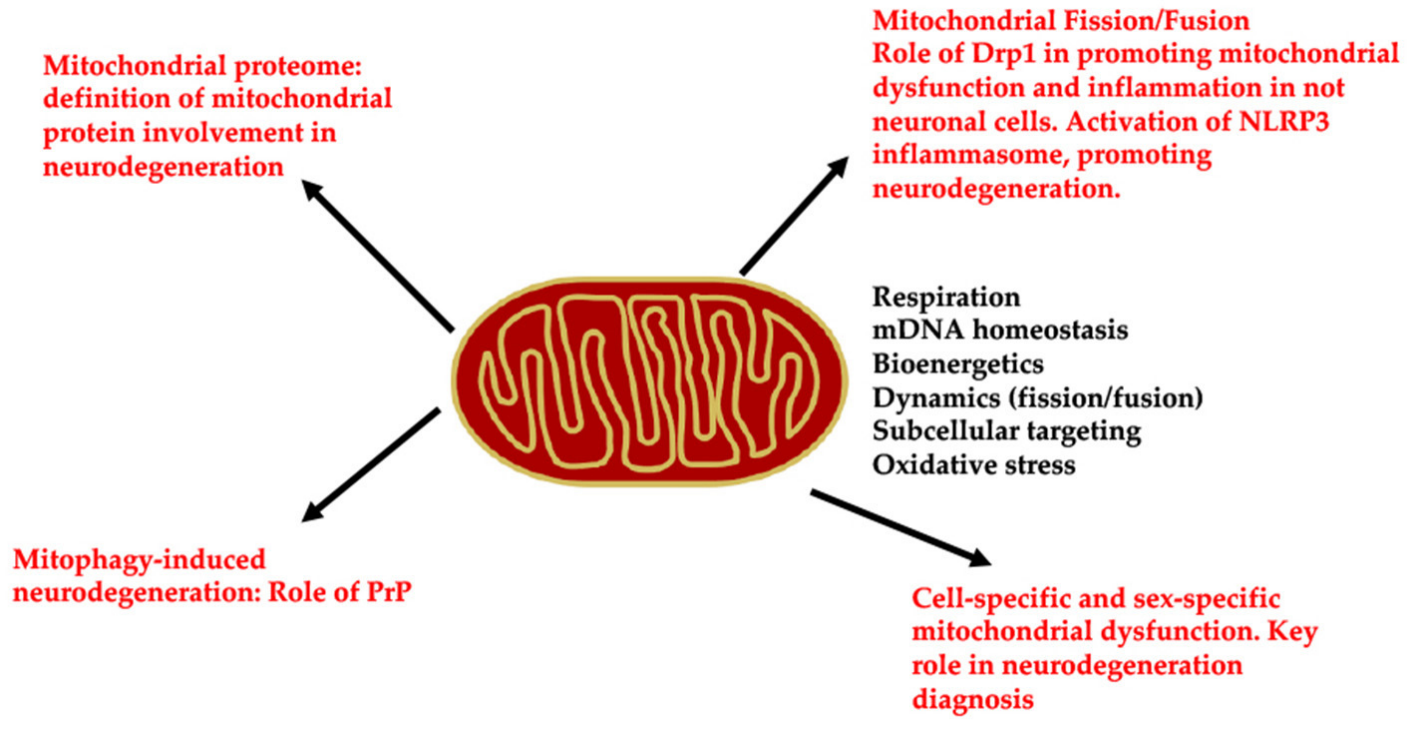
Schematic representation of the molecular pathways involved by mitochondrial dysfunction, leading to neurodegeneration: (i) n proteomic analysis to unveil the mitochondrial alterations in neurodegeneration; (ii) Drp1 regulation in mitochondrial fission/fusion and its alterations leading to neurodegeneration; (iii) cell specific and sex specific mitochondrial function and their alterations in neurodegeneration.

## Author contributions

LP: Writing – original draft, Writing – review and editing.
